# Preparing the healthcare workforce in South Africa for short-course rifampicin-resistant TB treatment: inter-professional training and task-sharing considerations

**DOI:** 10.1186/s12960-020-00552-1

**Published:** 2021-01-06

**Authors:** Jason E. Farley, Norbert Ndjeka, Khaya Mlandu, Kelly Lowensen, Keri Geiger, Yen Nguyen, Chakra Budhathoki, Paul D. Stamper

**Affiliations:** 1 The REACH Initiative, Johns Hopkins University School of Nursing, 855 N. Wolfe Street | Rangos Building Suite # 601, Mailbox #30, Baltimore, MD 21205 United States of America; 2 National Department of Health, CBD, Civitas Building, 222 Thabo Sehume St, Pretoria, 0001 South Africa

**Keywords:** Clinical, Primary health care, South Africa, Rifampicin-resistant, Tuberculosis, Multidrug-resistant, Nurse practitioner

## Abstract

**Background:**

Treatment for rifampicin-resistant *Mycobacterium tuberculosis* (RR-TB) is complex, however, shorter treatment, with newer antimicrobials are improving treatment outcomes. The South African National Department of Health (NDoH) recently accelerated the rollout of 9-month, all-oral, RR-TB short-course regimens. We sought to evaluate an inter-professional training program using pre-test and post-test performance of Professional Nurses (PNs), Advanced Practice Professional Nurses (APPNs) and Medical Officers (MOs) to inform: (a) training needs across cadres; (b) knowledge performance, by cadres; and (c) training differences in knowledge by nurse type.

**Methods:**

A 4-day didactic and case-based clinical decision support course for RR-TB regimens in South Africa (SA) was developed, reviewed and nationally accredited. Between February 2017 and July 2018, 12 training events were held. Clinicians who may initiate RR-TB treatment, specifically MOs and PN/APPNs with matched pre–post tests and demographic surveys were analyzed. Descriptive statistics are provided. Pre–post test evaluations included 25 evidence-based clinically related questions about RR-TB diagnosis, treatment, and care.

**Results:**

Participants (*N* = 842) participated in testing, and matched evaluations were received for 800 (95.0%) training participants. Demographic data were available for 793 (99.13%) participants, of whom 762 (96.1%) were MOs, or nurses, either PN or APPNs. Average correct response pre-test and post-test scores were 61.7% (range 7–24 correct responses) and 85.9% (range 12–25), respectively. Overall, 95.8% (730/762) of participants demonstrated improved knowledge. PNs improved on average 25% (6.22 points), whereas MOs improved 10% (2.89 points) with better mean test scores on both pre- and post-test (*p* < 0.000). APPNs performed the same as the MOs on post-test scores (*p* = NS).

**Conclusions:**

The inter-professional training program in short-course RR-TB treatment improved knowledge for participants. MOs had significantly greater pre-test scores. Of the nurses, APPNs outperformed other PNs, and performed equally to MOs on post-test scores, suggesting this advanced cadre of nurses might be the most appropriate to initiate and monitor treatment in close collaboration with MOs. All cadres of nurse reported the need for additional clinical training and mentoring prior to managing such patients.

## Background

New, shorter course treatment regimens for rifampicin-resistant *Mycobacterium tuberculosis* (RR-TB) are improving treatment outcomes in South Africa (SA) [1]. SA has boldly adopted an all-oral, 9–12 month short-course RR-TB treatment regimen, aligning with one option in the new World Health Organization (WHO) RR-TB treatment guidelines [2]. Initial RR-TB cohorts, in which up to 60–70% of patients are co-infected with human immunodeficiency virus (HIV) and include a high proportion of patients with extensively drug-resistant TB (XDR-TB), continue to demonstrate promising results [1, 1].

Since 2013, SA embarked on a plan to decentralize RR-TB treatment to primary care settings, many with nurse-led models of care. This nurse-led approach followed a task-sharing model, in which Professional Nurses (PNs) with advanced practice preparation (i.e., Advanced Practice Professional Nurses (APPN)) would initiate and manage patients with RR-TB and HIV co-infection. A Medical Officer (MO) was available for clinical consultation. A prospective cohort study evaluated this approach and demonstrated outcomes equivalent to that of an infectious disease-trained MO [4]. This observational study resulted in endorsement of a task-sharing approach through the National Strategic Plan for tuberculosis (TB), HIV and sexually transmitted infections [5], and the expansion of a nurse-led model of care to rural areas of the country. Since that time, investigators have identified that such community-based, nurse-led models may offer cost-effective alternatives to traditional hospital-based programs [6].

Introduction of the newer oral short-course regimen complicates the country’s decentralization of RR-TB treatment services to nurse-run primary care clinics. Three new agents (bedaquiline, linezolid and clofazimine) have been introduced into the regular regimen. Traditionally, nurses have not prescribed these agents, as their use was either restricted to XDR-TB patients or available only at tertiary care hospitals identified as national TB Centers of Excellence. The change also resulted in additional clinical monitoring requirements, with monthly ototoxicity monitoring being replaced by monthly electrocardiogram monitoring for QT prolongation. Further, due to a potent drug–drug interaction with efavirenz and bedaquiline, the standard antiretroviral (ART) regimen of fixed dose combination of efavirenz, tenofovir and emtricitabine must be switched for either nevirapine or a protease-inhibitor-based regimen. Each of these regimen modifications has been standardized into simplified algorithms to accommodate nurse-led approaches to care, yet correct treatment decisions for RR-TB is critical for successful patient management.

Understanding the need to ensure safe and convenient patient care for RR-TB, while making certain such care was scalable to all areas of the country, a structured educational training program in partnership with the SA National Department of Health (NDoH) was developed. In-service training is a first step required when implementing new clinical treatment guidelines and programs. Prior studies have demonstrated better clinical outcome measures with gains in knowledge and application of learned content to clinical management immediately after training. Confidence to provide effective care has also been demonstrated in studies that utilized an interactive, didactic and case-based approach to short-course training where participants have opportunities to get feedback from trainers and engaged in shared learning [7, 7]. Multiple studies from low- and middle-income countries have demonstrated that multi-day, face-to-face in-service trainings improve providers’ knowledge of TB disease and clinical treatment guidelines [9]. Evaluation of such training methods is essential, as governments determine the appropriate cadre of clinical workers to expand services. The purpose of this paper was to evaluate such a training and elucidate pre-test and post-test scores to guide recommendations for future training approaches.

## Methods

Under a collaborative agreement with the NDoH of SA, our team developed a certificate-based short-course for RR-TB management. The course was designed for healthcare workers (HCWs) in primary health care centers and TB settings, preparing them to plan and offer treatment for RR-TB care in the community. The program provided a detailed overview of clinical management, with special emphasis on treatment initiation at the community level. The course offered continuing professional development (CPD) accreditation from the South African Medical Association. The training events were held throughout South Africa and scheduled in accordance to NDoH and local departments’ needs from February 2017 through July 2018.

### Course structure

The course was organized into a 4-day, module-based design, with 3–4 modules per day as follows:

- Day 1 modules—key concepts and definitions; TB and TB/HIV epidemiology; latent vs active disease; differentiation of clinical, epidemiologic and microbiologic features of TB compared to RR-TB.
- Day 2 modules—RR-TB screening and diagnostics; baseline clinical evaluation; and treatment regimens.
- Day 3 modules—RR-TB and HIV co-infection; RR-TB and special populations (i.e., pregnancy, diabetes, substance use); community-based and patient-centered care approaches.
- Day 4 modules—integrated case study and participant case study presentations; infection control practices; RR-TB recording and reporting structure.

Each didactic module included case presentations, group-based interactions and/or facilitated discussion. The course faculty included MDR-TB scientists, MDR-TB and HIV-trained physicians and nurses, a nurse practitioner trained in MDR-TB treatment initiation, epidemiologist from the local department of health, as well as patients who successfully completed MDR-TB treatment.

### Course materials development

The curriculum was developed using SA National TB Guidelines [10], which were based on WHO recommendations [11], and related research [12, 13]. The course modules were organized to guide the participants through progressively more complicated didactic material followed by case-based application of the corresponding material. All patient scenarios were derived from actual TB, RR-TB or TB/HIV case situations from SA. A participant guidebook accompanied lectures to provide supplemental materials and a pocket guide offered quick reference to the salient clinical points. Each didactic lecture underwent three separate reviews: (1) assessment by the faculty and staff at The REACH Initiative of The Johns Hopkins University School of Nursing; (2) assessment by the NDoH; and, (3) assessment by the South African Medical Association for CPD credits. In each step of review, feedback was obtained with comments and corrections incorporated. Prior to launch, the NDoH adopted the training partners to utilize the approved training package.

### Participant selection

Any healthcare professional was eligible to attend the course as invited by the provincial TB leadership. Invitations were based on strategic needs of the province related to an intended goal of decentralization of treatment services to improve access to care. Participant selection also ensured that all trainings were interdisciplinary and included medical officers, pharmacists and nursing staff.

### Evaluation metrics

Before each training, participants completed a 25 question, multiple choice pre-test to determine knowledge of RR-TB. The pre- and post-test was designed by the RR-TB training team members based on content expertise and the SA clinical treatment guidelines. Prior to use, the instrument was assessed for face validity by the lead physician at the Drug-Resistant TB Program of the South Africa National Department of Health who is responsible for leading the national RR-TB guideline development process. Of the 25 questions, 10 were general knowledge questions about RR-TB and 15 questions required application of material by brief clinical case scenarios. At the conclusion of the training, the same questionnaire was administered to assess change in knowledge as a result of workshop participation. All participants were asked to provide basic demographic information. Qualitative course evaluations were anonymously collected using an open-ended questionnaire with five questions and two yes/no questions. The open-ended questionnaire addressed the programs overall rating, it’s strengths and weaknesses, as well as perceived needs of additional training with the final question giving participants the opportunity to provide additional comments.

### Ethical review

This training was implemented as part of public health practice involving educational tests with survey procedures. The Johns Hopkins Institutional Review Board reviewed and approved the project as exempt research (IRB00212633), which did not require consent. However, all participants received an overview letter with the instruction that their completion of the demographic form, pre- and post-test was voluntary and, if completed, was an authorization to evaluate the individual’s test score and demographic data.

### Data analysis

Data capture was performed using Excel (Microsoft, WA). Descriptive statistics and tests of strength of association (Chi-square, t-test) were calculated using Stata 15 (Stata Corporation, TX). Qualitative evaluations were open text and were reviewed for themes as to opportunities to improve along with additional training needs. Respondents offered open-ended responses to “What additional training do you need to initiate RR-TB treatment?” Only participants with completed demographic and matched pre–post tests were analyzed. The cadres of MOs and PN/APPNs were included in the analysis because they have the potential to initiate and/or support RR-TB treatment. The health care professionals excluded from the analysis because they did not identify with initiating patient treatment included: Academic Lecturer (*n* = 2), Clinical Associate (*n* = 1), Community Speech Pathologist (*n* = 1), Enrolled Nurse (*n* = 19), Epidemiologist (*n* = 1), and Pharmacist (*n* = 7). Analysis compared MOs to PNs, and further elucidated PNs, particularly APPNs identified as those having completed both the Advance Course Primary Health Care (PHC) and Short Course Training in Nurse Initiated Management of Antiretroviral Therapy (NIMART).

## Results

**Fig. 1:**
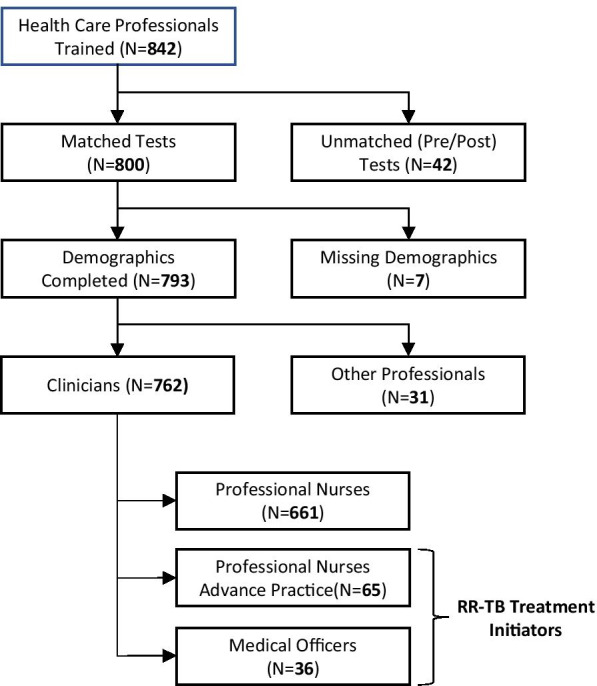
Population of trainees with demographic data and pre/post tests. This figure diagrams the selection of the healthcare workforce participants analyzed during the inter-professional training in South Africa

Twelve trainings including 842 healthcare professionals were conducted between February 6, 2017 and July 6, 2018 throughout SA. After exclusions, the 762 participants included for analysis had matched pre-tests and post-tests with complete data (Fig. 1). Of these, 661 (83.96%) were PNs without APPN training, and of these 555 (83.96%) were female (Table 1). Most PNs reported being between 35 and 44 years of age (38.58%), and the majority (84.57%) reported no prior TB experience. Sixty-five APPNs had completed preparations designating them as APPNs. The APPNs were also more likely to be female, older (majority reported age range between 45 and 54 years) and more likely to report some RR-TB experience (46.15%), than the PNs. Among the 36 MOs, the majority were male (61.11%), with the majority reporting an age range between 35 and 44 years, and having at least 6 months or more of RR-TB experience (61.11%).

**Table 1:** Participant characteristics (%) (*n* = 762 matched tests and participant demographics)

	Medical Officer (MO) (*N* = 36)	Advanced Practice Professional Nurse (APPN) (*N* = 65)	Professional Nurse (PN) (*N* = 661)	Total (*N* = 762)
Gender				
Female	14 (38.89)	46 (70.77)	555 (83.96)	615 (80.71)
Male	22 (61.11)	19 (29.23)	106 (16.04)	147 (19.29)
Age				
18–34 years	4 (11.11)	8 (12.31)	231 (34.95)	243 (31.89)
35–44 years	15 (41.67)	20 (30.77)	255 (38.58)	290 (38.06)
45–54 years	11 (30.55)	25 (38.46)	142 (21.48)	178 (23.36)
55+ years	6 (16.67)	12 (18.46)	33 (4.99)	51 (6.69)
RR-TB experience				
No experience	10 (27.78)	35 (53.85)	559 (84.57)	604 (79.27)
< 6 months	4 (11.11)	5 (7.69)	25 (3.78)	34 (4.46)
6 months–1 year	5 (13.89)	4 (6.15)	17 (2.57)	26 (3.41)
1 year–3 years	4 (11.11)	8 (12.31)	20 (3.03)	32 (4.20)
3 years–6 years	4 (11.11)	6 (9.23)	22 (3.33)	32 (4.20)
> 6 years	9 (25.00)	7 (10.77)	18 (2.72)	34 (4.46)

MOs (*N* = 36) performed better on both pre-test score and post-test score than all nurses, including both APPNs and PNs (*N* = 726) (mean pre-test, 17.30/25 [*p* < 0.000] and mean post-test, 22.13/25 [*p* = 0.0332]). Among APPNs, the mean pre-test score (18.08/25) was lower than the MO (*p* = 0.0003), yet the mean post-test score (22.37/25) were equivalent to MOs (*p* = 0.1214) (Table 2). The APPNs demonstrated a significant improvement between pre- and post-test scores (*p* = 0.0110) compared to the MOs.

**Table 2:** Comparison of pre- and post-test score means of medical officers to cadres of nurses (test)

Outcome variable	Medical Officer (MO) (*N* = 36)	Advance Practice Professional Nurse (APPN) (*N* = 65)		Professional Nurse (PN) (*N* = 661)	
	Mean (SD)	Mean (SD)	*T* (*p*-value)	Mean (SD)	*T* (*p*-value)
Pre-test score (0–25)	19.89 (2.80)	18.08 (2.27)	3.5305 (0.0006)	14.92 (2.78)	10.4559 (< 0.0000)
Post-test score (0–25)	22.78 (1.42)	22.37 (1.80)	1.1749 (0.2429)	21.33 (2.22)	3.8742 (0.0001)
Change score, difference	2.89 (2.54)	4.29 (3.01)	− 2.3660 (0.0199)	5.17 (3.17)	− 6.8456 (< 0.0000)
Relative change (%)	16.89 (19.57)	25.93% (20.22)	− 2.1763 (0.0319)	35.37% (28.93)	− 6.0078 (< 0.0000)

*N* number, *SD* standard deviation

Written qualitative evaluation data were available from all but 42 participants (2 physicians, 2 APPNs and 38 nurses); not all participants wrote answers to every question. The following additional training themes were reported by more than 10% of participants, regardless of clinical preparation: case studies were helpful (*n* = 232), but more case studies (*n* = 87) and supervised clinical application post-training (*n* = 127), were particularly noted in nurse participants.

## Discussion

This report describes the impact of inter-professional training on knowledge for RR-TB clinical care. We found that all cadres of nurses scored lower on baseline knowledge and reported less RR-TB experience (< 6 M experience) than MOs. APPNs, however, improved their post-test scores demonstrating knowledge post-training equivalents to MOs, despite having reported less experience caring for patients with RR-TB. This training is an initial step in our understanding of the potential opportunity to task-share the management of RR-TB using short-course regimens. While it is not possible to extrapolate findings on pre–post-test knowledge to safe and effective clinical care, this data can be used to guide decision-making on the cadre of nurse best suited for such care. Focusing time, energy and resources through short-course training of Professional Nurses with advanced practice training (i.e., APPNs) may offer one solution to the human resource challenges that limit access to care in some settings.

To date, only one small cohort of 197 patients was used to evaluate a nurse-led RR-TB program [4]. In this study of 24-month injection-based treatment in SA, patients initiated and managed by an APPN demonstrated outcomes equivalent to the MOs at a single peri-urban site. Overall, the APPN patients’ outcomes were better than the national average for the country during the same period of time [4]. The APPN had access to a MO who served as the clinical mentor and a research team of highly trained RR-TB clinical experts. It is unclear whether these findings could be replicated in a real-world scale-up of such treatment models. It is also unclear the manner in which shorter treatment regimens with more potent antimicrobials would impact this outcome. While it is theorized that the newer short-course, all-oral, RR-TB regimen will improve adherence and reduce loss to follow-up, the treatment-related adverse events [14], drug–drug interactions [15] and pill burden [16] of an all-oral regimen may complicate treatment needs. Despite these challenges, data from SA and other sub-Saharan African countries on task-sharing between MOs and nurses are clear for both HIV [17, 18] and drug-susceptible TB [19]—outcomes are equivalent. The evidence thus far for RR-TB suggests that the APPN cadre of nurse may offer a suitable and complementary alternative to models that rely solely on MOs, however more evidence is clearly needed.

The inter-professional nature of the training program was designed with intent and based on prevailing evidence suggesting three core elements are required—didactic training, simulation/case studies and community experience [20]. In this program, our “community experience” was represented through former RR-TB patients as training facilitator, who communicated the ‘lived experience’ of surviving RR-TB as well as the influence of the healthcare team on their success. Drawing from the inter-professional literature and our prior experience with discipline-specific training programs in RR-TB, our team was keenly aware that MOs often saw the psychosocial aspects of the training as unnecessary, while general nurses most often did not want an exhaustive understanding of treatment decision-making. To overcome these barriers, we ensured that all case-based applications sought to engage the learners in the comprehensive management of the patient’s care. This required the groups to function as teams in the small break-out sessions where cases were being applied, thus facilitating insight into the clinical decision-making of each cadre. Although we did not measure participant perceptions of this approach directly, our training evaluations were overwhelmingly positive about the team case-based approach, a finding consistent with available literature on learner, receptivity, to inter-professional education in the health professions [21, 22].

This evaluation has important limitations that future research should seek to explore. As our pre- and post-test measured only immediate knowledge gain, we are unable to determine knowledge retention in this context. APPNs, by their own account, still require additional mentored clinical training. This review did not explore the clinical aspects of their training, nor outcomes of such training on patient care. The effective length and duration of effective supervision of such training is also not known. Further, APPNs are fewer in SA than the PN cadre. The trend in HIV care has been to engage a PN without advanced practice training in a short-course for initiation and management of ART. This approach has proven highly effective for the scale-up of ART throughout the country, yet is based on limited HIV treatments and an algorithm approach that guides decision-making [23, 24]. The treatment regimen for RR-TB is also highly regimented, however, the laboratory and clinical monitoring are more complex and require changes to the HIV treatment regimen in many patients. Finally, as the implications of non-adherence are profound, were APPNs to integrate RR-TB treatment into a primary care setting, services that support adherence, engagement and retention in care would be essential [25].

## Conclusion

In this pre–post-test evaluation of didactic and case-based inter-professional training for short-course RR-TB treatment, MOs had greater RR-TB experience and outperformed nurses in pre-test evaluations. After the 4-day training, APPNs scored equally as well as MOs on post-test, despite having less experience. Both APPNs and MOs outperformed PNs. Focusing time, energy and resources through short-course training of Professional Nurses with advanced practice training (i.e., APPNs) may offer one solution to the human resource challenges that limit access to care in some settings. Future RR-TB trainings should combine short-course training followed by an immediate period of supervised clinical training with serial measurements for the development of clinical competency, patient safety and knowledge retention.

## Data Availability

Not applicable as results are published. Should readers require additional information, the authors will provide the available data upon request.

## Abbreviations

APPNs: Advanced Practice Professional Nurses
CPD: Continuing professional development
HCWs: Healthcare workers
HIV: Human immunodeficiency virus
MO: Medical Officer
NDoH: National Department of Health
PHC: Primary Health Care
PNs: Professional Nurses
RR-TB: Rifampicin-resistant *Mycobacterium tuberculosis*
SA: South Africa
TB: Tuberculosis
WHO: World Health Organization
XDR-TB: Extensively drug-resistant TB
